# Stated and revealed preferences for HIV testing: can oral self-testing help to increase uptake amongst truck drivers in Kenya?

**DOI:** 10.1186/s12889-018-6122-1

**Published:** 2018-11-06

**Authors:** Michael Strauss, Gavin George, Joanne E. Mantell, Matthew L. Romo, Eva Mwai, Eston N. Nyaga, Jacob O. Odhiambo, Kaymarlin Govender, Elizabeth A. Kelvin

**Affiliations:** 1 Health Economics and HIV and AIDS Research Division, University of KwaZulu-Natal, 4th Floor J-Block, University of KwaZulu-Natal Westville Campus, University Drive, Durban, 4041 South Africa; 2 Division of Gender, Sexuality and Health, Department of Psychiatry, HIV Center for Clinical and Behavioral Studies, New York State Psychiatric Institute & Columbia University, 1051 Riverside Drive, New York, NY 10032 USA; 3 Department of Epidemiology and Biostatistics, CUNY Graduate School of Public Health and Health Policy and Institute for Implementation Science in Population Health, City University of New York, 55 West 125th Street, New York, NY 10027 USA; 4 North Star Alliance, PO Box 165, Nairobi, 00202 Kenya

**Keywords:** HIV, HIV testing, Truck drivers, Discrete choice experiment, Kenya

## Abstract

**Background:**

Long-distance truck drivers in Africa are particularly at risk of HIV acquisition and offering self-testing could help increase testing coverage in this hard-to-reach population. The aims of this study are twofold: (1) to examine the preference structures of truck drivers in Kenya regarding HIV testing service delivery models and what they mean for the roll-out of HIV self-testing, and (2) to compare the preference data collected from a hypothetical discrete choice experiment with the actual choices made by participants in the intervention arm of a randomised controlled trial (RCT) who were offered HIV testing choices.

**Methods:**

Using data from 150 truck drivers, this paper examines whether the stated preferences regarding HIV testing in a discrete choice experiment predict the actual test selected when offered HIV testing choices. Conditional logit models were used for main effects analysis and stratified models were run by HIV testing choices made in the trial to assess if the attributes preferred differed by test chosen.

**Results:**

The strongest driver of stated preference among all participants was cost. However, two preferences diverged between those who actually chose self-testing in the RCT and those who chose a provider administered test: the type of test (*p* < 0.001) and the type of counselling (p = 0.003). Self-testers preferred oral-testing to finger-prick testing (OR 1.26 *p* = 0.005), while non-self-testers preferred finger-prick testing (OR 0.56 *p* < 0.001). Non-self-testers preferred in-person counselling to telephonic counselling (OR 0.64 *p* < 0.001), while self-testers were indifferent to type of counselling. Preferences in both groups regarding who administered the test were not significant.

**Conclusions:**

We found stated preference structures helped explain the actual choices participants made regarding the type of HIV testing they accepted. Offering oral testing may be an effective strategy for increasing willingness to test among certain groups of truck drivers. However, the importance of in-person counselling and support, and concern that an oral test cannot detect HIV infection may mean that continuing to offer finger-prick testing at roadside wellness centres will best align with the preferences of those already attending these facilities. More research is needed to explore whether who administers the HIV test (provider versus self) makes any difference.

**Trial registration:**

This trial is registered with the Registry for International Development Impact Evaluations (RIDE ID#55847d64a454f).

## Background

Delivering HIV testing and counselling (HTC) services to high-risk populations is important for early detection, prevention and monitoring of HIV, and is a cost-effective approach to reaching the UNAIDS 90–90-90 targets due, in part, to the higher HIV infection rates relative to the general population [1–3]. Long-distance truck drivers in Africa are one such population because truckers often have high levels of involvement with female sex workers [2, 4], engage in multiple concurrent sexual partnerships [5–8] and report inconsistent condom use. Levels of HIV testing among truck drivers have been historically low [3], and access to services is often limited [2, 9, 10].

The trucking industry is one of the biggest in east and southern Africa and has grown substantially in recent years. It is estimated that there are over 1500 trucking companies based in Kenya, where this study was conducted, operating more than 17,000 trucks [11]. The major trucking route through Kenya includes a significant portion of the Northern and Central Corridors through east and southern Africa, thus there are many long-distance truck drivers from other countries in the region who travel through Kenya. The North Star Alliance operates a network of roadside wellness centres in east and southern Africa that provide primary and secondary healthcare services (including HTC) to long-distance truck drivers as well as the sex workers and roadside community residents who provide services to them [12]. In 2017, the North Star Alliance facilities served almost 200,000 clients with primary healthcare, STI, HIV, malaria and tuberculosis services [12].

Self-testing for HIV may be strategically important for increasing HIV testing uptake among truck drivers, and other high-risk or mobile populations [13]. Introducing HIV self-testing (HST) could decrease the burden on the health care system, especially in terms of human resources, and the additional test options available to clients may increase HIV testing coverage [13–15]. However, although people may report that health interventions are acceptable, this may not necessarily translate into high levels of uptake, both for individuals and at the population level (for example, although studies found high levels of acceptability for HPV vaccination in Kenya, actual uptake remained low [16]). Although HST may be broadly acceptable, some clients could still prefer existing services, or at least prefer some of the characteristics of existing HIV testing models. Thus, analysis of the preference structures underlying testing choices is important for understanding how increasing testing options could translate into increased uptake of HIV testing.

This study examines the preferences and drivers of choice regarding HIV testing service delivery characteristics using stated preference (SP) data collected in a discrete choice experiment (DCE) where participants made choices between different hypothetical HIV testing service delivery models. We examine these preference structures in relation to revealed preference (RP) data relating to the actual HIV test chosen by participants who were offered HIV testing choices in the intervention arm of a randomised controlled trial (RCT). There are several potential strengths and limitations of both SP and RP methods. Some authors have argued that SP data are unreliable because of the hypothetical nature of choice experiments and the lack of linkages with real-world consequences [17–19]. However, the design of SP experiments may determine the underlying preference structures that drive choices, which are often unobservable when participants make real choices in either experimental or real-world environments [17]. Also, it may not be feasible to offer a large variety of choices in the real world and SP may be the only feasible way to assess which characteristics are preferred – which can help to inform the design of service delivery models that better align with client preferences and increase demand. While RP data can be more reliable for understanding preferences because they reflect actual choices made by participants, within the context of an RCT, these choices may be subject to biases related to the research setting and participants’ awareness that they are being studied [20, 21], which may help explain some of the challenges when translating the positive results of an RCT into scaled-up real-world programmes [22]. Numerous highly effective interventions have not gained sufficient traction in some settings due to insufficient capacity of the health system to provide services, or a lack of demand for services, failing to reach the coverage targets required to impact population health. One example is the voluntary medical male circumcision programme in east and southern Africa where, in spite of successes in some countries, many others are falling short of the targets set in 2011 [23, 24].

Therefore, the aims of this analysis are twofold. The first is a methodological consideration of how SP data from a DCE can be used to predict the actual choices made by participants in an RCT (their RP) both at baseline when in the clinic from which they were recruited and over a six-month follow-up period, which may be more reflective of a real-world service delivery setting than directly following recruitment into a study. The integration of SP and RP data is an important under-studied gap in the literature [25, 26]. Second, we look more broadly at preference structures and interpret what these results mean for the potential roll-out of HST and demand amongst truck drivers in Kenya, focusing particularly on the characteristics of testing delivery models that may facilitate or act as barriers to testing uptake for truck drivers.

## Methods

### Theoretical framework

Two economic theories underpin DCEs. The first, Lancaster’s Theory of Consumer Choice [17], states that consumers will make choices to maximise utility from consumption based on the utility derived from the characteristics (or attributes) of goods or services [27]. The implication is that preferences relating to the attributes of a service drive choice, rather than the service as a whole. In the context of HIV testing, this means that preferences regarding the characteristics of the service delivery model may significantly influence the decision to test. Since consumers make choices to maximise utility, we assume that when a person chooses one type of service delivery model over another, it is because they derive more utility from the combination of characteristics of their choice.

Second, Random Utility Theory suggests that the utility derived from choice is attributable to both a systematic and a random component and forms the basis for analysis of data on choices [28]. The systematic component comes from observable characteristics of the good or service, which is made up of different attributes and the individual making the choice. The random component estimates the effect any unobservable or unexplainable factors may contribute to overall utility, as well as measurement or specification error (16–17). Thus, total utility *U*_*ij*_ depends on the utility derived from each of the attributes X_*ij*_ such that

$$ {U}_{ij}={\beta}_{ij}{X}_{ij}+{\varepsilon}_{ij} $$

where *X*_*ij*_ is a vector of the attributes and levels (see Table 1), and *ε*_*ij*_ is the random component for every individual *i* making choice *j*.

**Table 1:** Attributes and Levels

Attribute	Levels
Type of counselling	1. In-person counselling (reference category)
	2. Telephonic counselling
Who administers the test	1. Provider-administered (reference category)
	2. Self-administered
Type of test	1. Finger-prick blood test (reference category)
	2. Oral mouth-swab test
Location	1. At a roadside clinic (reference category)
	2. At a clinic near home
	3. At the company office
	4. At home
Cost	1. Free (reference category)
	2. You pay 250 Kenyan Shillings (US$2.50)
	3. You pay 300 Kenyan Shillings (US$3.00)
	4. We pay you 350 Kenyan Shillings (US$3.50)
Time	1. 90 min (reference category)
	2. 20 min
	3. 40 min
	4. 3 h

### Study setting and sample size

This study was conducted at two of the eight North Star Alliance roadside wellness centres in Kenya which are located in Nakuru county, one of the counties in Kenya with the highest HIV prevalence [29, 30]. Each of these two clinics serves approximately 400 clients a week, about 30% of whom are truck drivers. Participants were recruited into an RCT from the clinic waiting rooms from October 2015 to December 2015. The RCT aimed to assess the impact of adding HST choices in the standard HIV testing program on testing uptake among truck drivers. The trial methods and results are presented in detail elsewhere [31, 32], but here we briefly describe the aspects that are relevant to this paper. Eligibility criteria for inclusion in the trial were: (1) at least 18 years old; (2) male (given that the industry almost entirely comprises men); (3) employed as a truck driver; (4) primary residence in Kenya; (5) able to speak English or Kiswahili; (6) self-reported HIV-negative or unknown HIV status; (7) ability to sign the consent form; and (8) ability to receive a small compensation of 270 Kenyan shillings (KES), approximately US$2.70, for each of the two baseline interviews and 360 KES, approximately US$3.60, for completion of the follow-up interview using MPesa – a cell phone-based money transfer system widely used in Kenya.

In the first phase of the study (T1), participants completed a baseline interview about demographic characteristics, HTC experiences, sexual history and risk behaviours, and the DCE module of questions. Participants were then randomly assigned to one of two arms – only participants in the intervention arm were offered HIV self-testing in addition to the standard provider-administered test, whereas those in the control arm were only offered the provider-administered test. Because this paper focuses on how responses on a DCE predict HIV testing choices when offered, we included only those in the intervention arm in these analyses. Participants in the intervention arm were first offered a choice between supervised oral HST and provider-administered finger-prick testing (both options were for testing in the clinic with a provider present). Those who refused both of these in-clinic testing options were then offered an HST kit for home use with phone-based post-test counselling [31]. Following HIV testing or test refusal, participants completed the second baseline questionnaire in which they answered questions about their testing experiences and the reasons behind their HIV testing choice. Participants in the intervention arm could also collect an oral HST kit for self-use in any of the eight North Star Alliance clinics in Kenya during a six-month follow-up period. At three months post-baseline, participants in the intervention arm were sent an SMS reminding them that HIV self-test kits were available at North Star clinics. Participants were interviewed again at six months (T2) over the phone about whether they had tested for HIV in the past 6 months and, if so, what HIV test (self-test or provider administered-test) they used and reasons for their choices.

A total of 150 of the 305 RCT participants were randomised to the intervention arm and are included in these analyses. This satisfied the minimum sample size of 125 needed for the DCE, calculated as:

$$ N\ge 500\frac{L}{SJ} $$

where L is the maximum number of levels for any attribute, S is the number of alternatives in each choice set, and J is the number of choice sets presented to each participant [33]. Empirical evidence suggests that as few as 20 participants per version of the questionnaire will lead to reliable model estimates [34].

### Testing attributes included in the DCE

Six attributes of HIV testing within the control of service providers were selected for inclusion in the DCE choice sets (see Table 1), based on discussions with key personnel at North Star clinics and other DCE studies on HIV testing [35]. Two levels in each attribute were selected to capture key differences between HST and the standard provider-administered testing offered at North Star clinics for type of counselling (in-person versus telephonic), who administers the test (provider versus self), and type of test (oral versus blood). For location, four levels (roadside clinic, clinic near home, company office, home) were examined to capture different levels of convenience and privacy. Four levels each for the cost (ranging from a payment of the equivalent of US$3.00, to free, to receipt of a payment of US$3.50) and time (ranging from 20 min to 3 h) captured variation that would reflect strength of preferences.

A fractional factorial design of 32 choice sets, each containing two unlabelled alternatives with different combinations of levels of all attributes, were designed by generating an orthogonal main effects plan for the first alternative in each choice. The second alternative in each choice set was generated by systematically adding one level (cyclically) to the level that appeared in the first choice set, resulting in an optimal design according to D-efficiency criteria and in line with the principles of efficient designs [36, 37]. Fieldworkers were trained and used scripted instructions to present choices to participants in one-on-one interviews, using laminated cards with pictures and words (both in English and Kiswahili) to explain choice options. Additional details on the DCE design are documented elsewhere [38].

### Analysis strategy and model estimation

A descriptive analysis of the choices made by enrolled participants in the intervention arm who were offered HIV testing choices at both T1 and T2 was conducted, looking at the number and percentage of participants who selected different methods of testing (or declined testing) and using a flow diagram to distinguish between the choices offered and the choices made at both T1 and T2. Associations between choices made at T1 and at T2 were tested for significance using Fisher’s exact test to compare both the decision to test or not to test and, for those who accepted testing either at T1 or T2 (or both), the decision about which type of test was selected.

We then looked at the preferences indicated in the DCE overall and by choices made in the RP at T1 and T2 in order to examine the association between the preference structures from the DCE and the actual choices made in the RCT and determine the attributes driving RP related to HST.

To estimate the main effects, dummy variables were created for each level of each attribute. The reference scenario was selected based on the typical HTC service clients would receive at a North Star clinic (see Table 1). A conditional (fixed effects) logit model was used for estimation of parameters:

$$ {\mathit{\Pr}}_{ij}=\frac{\exp \left(\beta {X}_{ij}\right)}{\sum_{k=1}^K\exp \left(\beta {X}_{ik}\right)}, $$

for all alternatives, K in the choice set

where *Pr*_*ij*_ is the probability of participant *i* choosing alternative *j* in each binary set of alternatives *K*, *β* is a column vector of parameter estimates associated with *X*_*ij*_, which is a row vector of the levels of the attributes in alternative *j* chosen by participant *i* [39]. Conditional logit models have been used successfully in this way in many other studies [25, 26, 35, 40], and assumptions are well aligned to the experimental design of this DCE [38]. In this type of DCE analysis, the dependent variable (i.e. which option out of two was selected by participants) is not a key consideration for the results of the estimation because both options represent an HIV test, but in each case the characteristics of the test varied. Thus, the actual choice (Option A or Option B) is irrelevant for this analysis, but the drivers of that choice provide insights into the underlying preference structures, estimated by the coefficients of the attribute levels in the model – in this paper reported as odds ratios against the reference attribute levels.

To understand how preference structures in the DCE analysis might explain the actual choices made, we assessed a series of models. Model 1 included all participants in the intervention for an assessment of the average preferences from the DCE in that group. Model 2 used the same model but stratified on HIV test selected at baseline (self-tested at baseline or provider-administered test; those who chose not to test were excluded from this analysis). Model 3 was stratified by HIV test used during the 6-month follow-up period (self-test collected from the clinic or a provider-administered test; those who chose not to test were excluded from the analysis), and Model 4 looked at homogeneity of test selected at both baseline and follow-up (both tests were provider-administered or both were self-administered; again, those who chose not to test were excluded from the analysis). Confidence intervals of 95% were used to assess statistical significance in the main effects and stratified models, and estimates were judged to be statistically significantly different between groups in the stratified analyses if 95% confidence intervals did not overlap.

There are few examples of studies that have combined SP and RP data in the same model, where the attributes and levels found in the RPs could be compared with the preferred attributes and levels contained in the hypothetical choices made by participants in the DCE [19, 26]. In this study, the SP data could be used to explain the actual choices made by participants, as well as provide additional insight into the drivers of those choices, as the RP data were only able to capture some of the variation with respect to the characteristics of HIV testing. For example, at T1, there was no variation depending on the type of test participants were offered with respect to the attributes of time and cost; and for location and the type of counselling offered, there was only variation for those who first declined both provider-administered finger-prick testing and supervised self-testing. Further, participants were not given an “opt-out” option in any of the DCE choices (as they were in the RP scenario in which they could refuse HIV testing altogether) because the DCE aimed to capture variation in preferences rather than estimate overall expected use of a particular type of testing, a design feature that increases the explanatory power of the DCE regarding the actual choices participants made, reducing biases introduced by participants opting out consistently because of choice complexity [41].

### Ethics

The study procedures were approved by the City University of New York Institutional Review Board, the Kenya Medical Research Institute Ethics Committee, and the University of KwaZulu-Natal Biomedical Ethics Committee in South Africa.

## Results

### Description of participants

Almost all participants (96.6%) reported being sexually active in the past six months. About half (48%) reported having regular partners along their trucking route in addition to a wife or girlfriend at home, and half (50%) reported having paid for sex in the past six months. Only 11.3% reported always using condoms during sex in the past six months. Most of the participants (90%) had tested for HIV at least once previously with a mean time since last test of 1.1 years (SD = 1.9 years). Many of the participants (40.7%) reported that they came to the clinic on the day they were recruited into the study specifically for an HIV test; half had tested previously at a North Star roadside wellness centre.

**Fig. 1:**
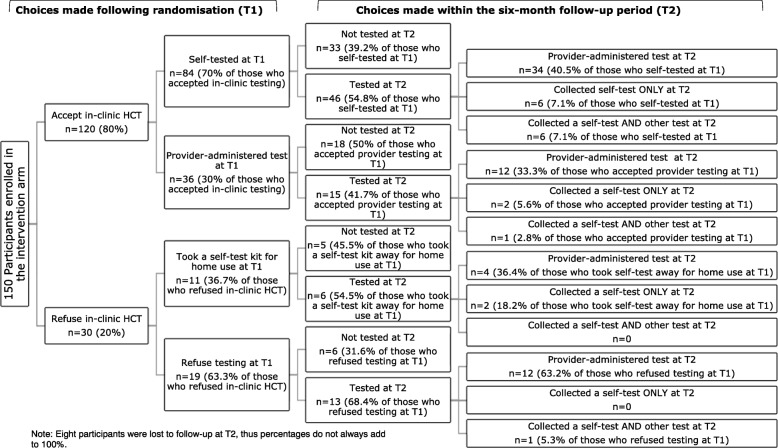
Flow of participant choices at T1 and T2. Note: Eight participants were lost to follow-up at T2, thus percentages do not always add to 100%

Figure 1 shows the flow of participants in the RCT following randomisation into the intervention arm and choices made at T1 and T2. Overall, uptake of HTC was higher at T1 than at T2 and the proportion of participants who accepted testing at T2 (self-testing or provider-administered testing) did not differ substantially depending on the choice made at T1.

Of the 84 participants who self-tested at the facility at T1, 34 participants (40.5%) accessed a provider-administered test by T2, 33 participants (39.2%) did not test during the follow-up period, six (7.1%) returned for a self-test by T2, and another six (7.1%) used both a self-test and a provider-administered finger-prick test during the follow-up period (i.e. they tested more than once with different HIV tests over the 6-month period). Of the 11 participants who refused in-clinic testing but took a self-test home at T1, only two (18.2%) returned for self-testing during follow-up (T2), while four (36.4%) returned for a provider-administered finger-prick test during follow-up (T2), and five (45.5%) did not test by T2. Of the 36 participants who selected a provider-administered test at T1, 12 participants (33.3%) accessed a provider-administered test by T2, 18 (50%) did not return for testing during follow-up, two participants (5.6%) returned for a self-test by T2, and one person (2.8%) used both a self-test and a provider-administered test during the follow-up period.

**Fig. 2:**
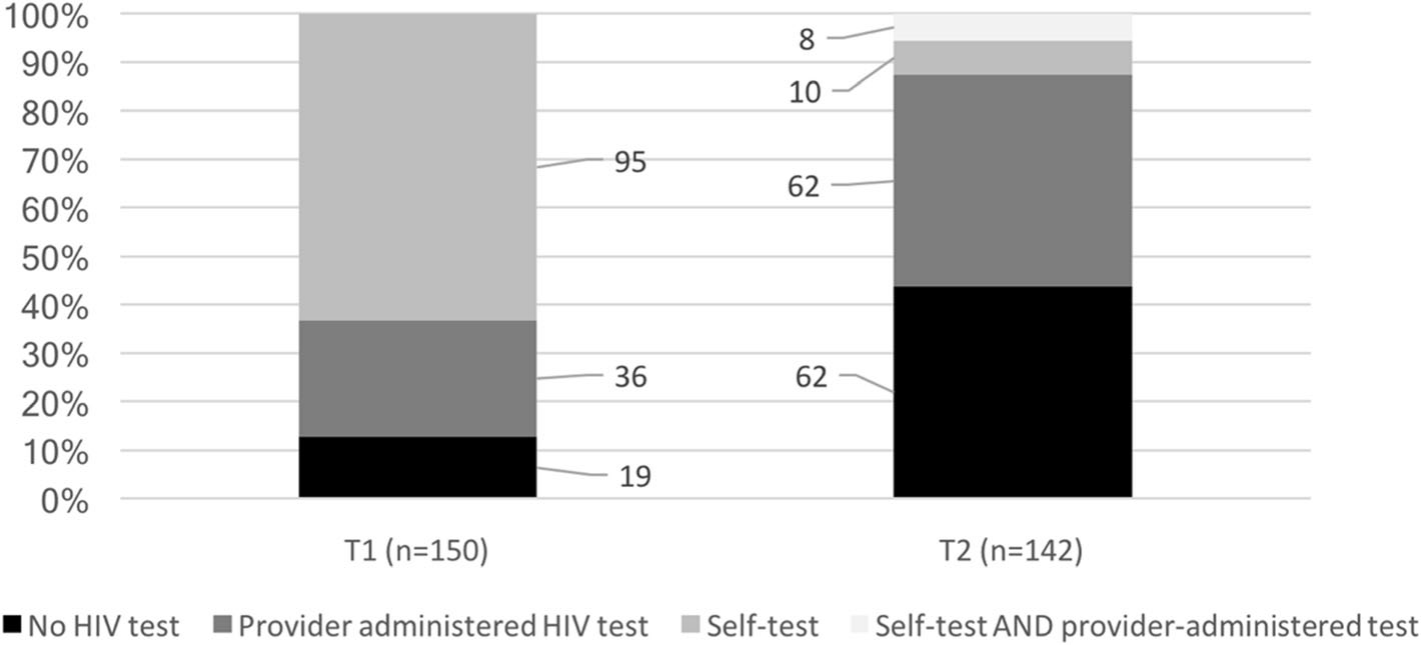
Testing choices at T1 and T2. Note: Eight participants (6%) were lost to follow-up at T2

Figure 2 shows the number and percentage of clients who tested at T1 in comparison to those that returned for testing within the six-month follow-up period. Overall, a smaller percentage of participants tested at T2 (56% compared to 87% at T1), with far fewer opting for HST (13% compared to 63% at T1).

### DCE results

#### Time 1

**Fig. 3:**
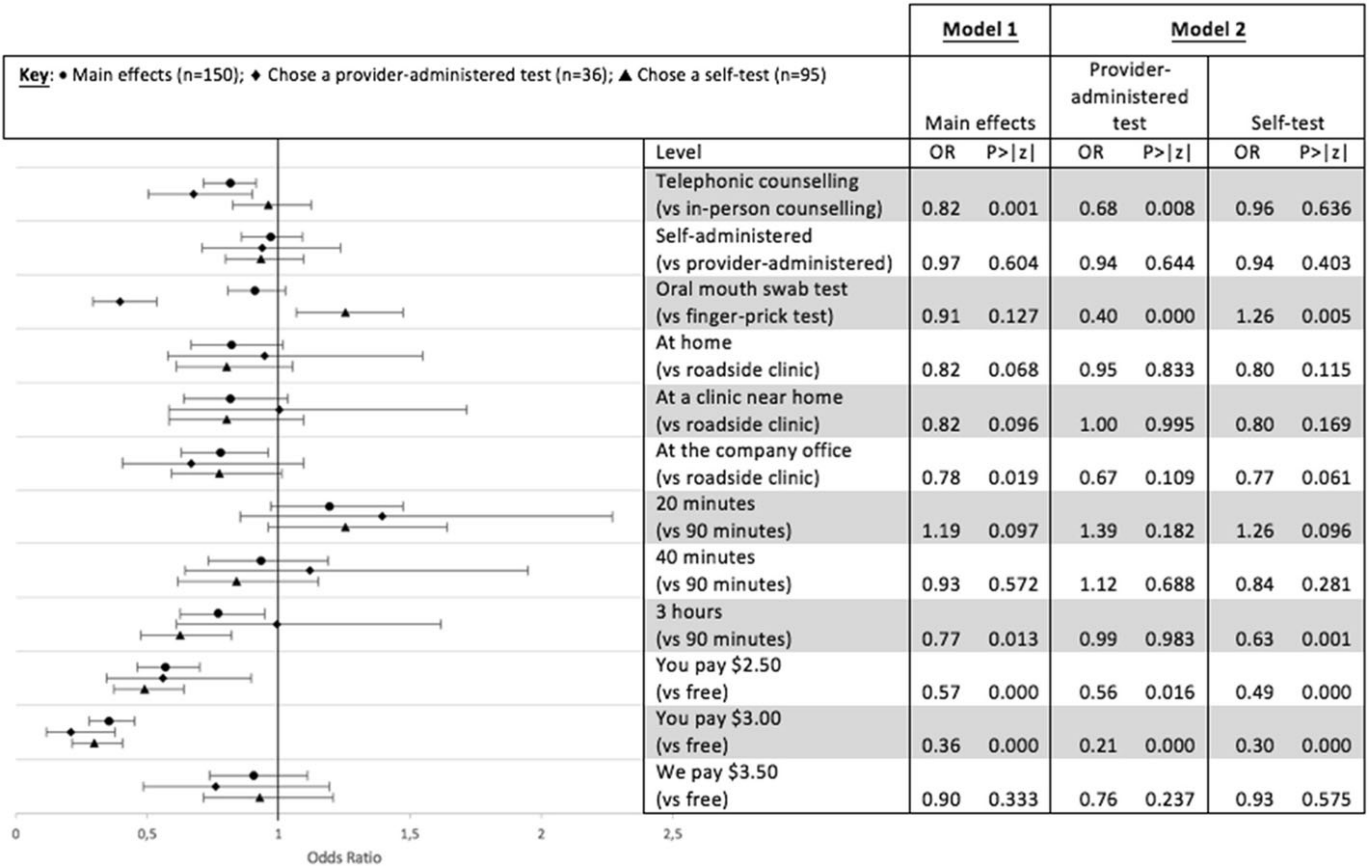
DCE results – main effects and stratified analysis by test selected at T1. Model 1 shows main effects results and Model 2 shows the results stratified by the type of test chosen at T1. Odds ratios and *p* -values are shown in the table and the figure presents odds ratios with 95% confidence intervals

The results from the main effects model shown in Fig. 3 suggest an overall preference for the reference category characteristics of testing among participants in the intervention arm (modelled on the North Star clinics HIV testing guidelines). Participants on average preferred in-person counselling over telephonic counselling (OR 0.82 *p* = 0.001) and testing at a roadside clinic over testing at the company office (OR 0.78 *p* = 0.019), but they were indifferent between testing at home or at a clinic near home versus a roadside clinic. Participants preferred the reference characteristic of 90 min over the 3 h for the HIV testing process (OR 0.77 *p* = 0.013); however, they were indifferent regarding 20 or 40 versus 90 min. The strongest driver of choice was cost, with a small fee of US$ 2.50 significantly reducing the odds of choosing a test (OR 0.57 *p* < 0.001) and a US$3.00 fee having the greatest effect on decreasing the odds of choosing a test (OR 0.36 *p* < 0.001) of any characteristic. There was no preference between the offer of a small incentive of US$3.50 versus a free test (OR 0.9 *p* = 0.333), suggesting that providing an incentive would be unlikely to increase demand. In the sample as a whole, participants were also seemingly indifferent between provider-administered HIV testing and self-administered testing (*p* = 0.97) and between an oral- versus blood-based test (*p* = 0.91) – although the stratified analyses presented below showed differences between groups.

In the analysis stratified by the test selected at T1, we found significant differences (95% confidence intervals do not overlap) in preferences between those who chose a self-test at T1 and those who selected a provider administered test at T1 in one key attribute: the type of test. Participants who chose HST preferred an oral test to a finger-prick test (OR 1.26 *p* = 0.005), while those who chose a provider administered test preferred a finger-prick test over an oral test (OR 0.56 *p* < 0.001).Further, we found that participants who chose to self-test were indifferent between telephonic and in-person counselling (note counselling for those who took a test kit for home use was over the phone), while those who chose a provider administered test preferred in-person rather than telephonic counselling (OR 0.64 *p* < 0.001).

#### Times 1 and 2

The results presented in Table 2 show the preference structures from the DCE analysis from Model 3 (stratified by choices made at T2) and Model 4 (stratified by choices made at both T1 and T2). The results of both Model 3 for those choosing to self-test at T2, and Model 4 for those choosing to self-test at both T1 and T2, found no significant differences in DCE attribute preferences (likely due to the small sample size in these strata) and therefore are not shown in the table. The results for Model 3 show the stratification for those choosing a provider-administered test at T2, and in Model 4, for those choosing a provider-administered test at both T1 and T2.

**Table 2:** DCE Results – Preferences of Participants who Returned for Provider-administered testing at T2 (Model 3); and who Consistently Selected Provider-administered Testing at T1 and T2 (Model 4)

	Model 3 Provider-administered test selected at T2 (*n* = 62)			Model 4 Provider-administered test selected at T1 and T2 (*n* = 12)		
	OR	P > |z|	95% CI	OR	P > |z|	95% CI
Telephonic counselling (vs in-person counselling ^a^ )	0.72	0.001	(0.60, 0.87)	0.35	0.001	(0.19, 0.65)
Self-administered (vs provider-administered)	0.95	0.574	(0.78, 1.14)	0.58	0.139	(0.29, 1.19)
Oral mouth swab test (vs finger-prick test)	0.83	0.054	(0.69, 1.00)	0.17	0.000	(0.08, 0.36)
At home (vs roadside clinic)	0.82	0.225	(0.59, 1.13)	0.98	0.968	(0.30, 3.22)
At a clinic near home (vs roadside clinic)	0.80	0.257	(0.55, 1.17)	2.01	0.328	(0.50, 8.10)
At the company office (vs roadside clinic)	0.77	0.113	(0.55, 1.06)	0.72	0.580	(0.22, 2.34)
20 min (vs 90 min)	0.91	0.568	(0.66, 1.26)	2.12	0.184	(0.70, 6.41)
40 min (vs 90 min)	0.93	0.707	(0.64, 1.35)	1.64	0.429	(0.48, 5.56)
3 h (vs 90 min)	0.81	0.209	(0.59, 1.12)	0.70	0.519	(0.24, 2.04)
You pay $2.50 (vs free)	0.60	0.002	(0.44, 0.83)	0.69	0.518	(0.22, 2.13)
You pay $3.00 (vs free)	0.40	0.000	(0.27, 0.58)	0.44	0.242	(0.11, 1.73)
We pay $3.50 (vs free)	0.92	0.623	(0.67, 1.27)	1.32	0.550	(0.53, 3.30)

^a^ Reference categories shown in brackets

Note: Preferences for those choosing to self-test at T2 in Model 3, and those choosing to self-test at both T1 and T2 in Model 4 were not significant in any of the attribute levels and are thus not presented in this table

The findings from this analysis show similar preference structures to those shown among participants who chose provider-administered testing at T1. Importantly, the direction of preferences regarding the type of counselling and the type of test remained the same in these groups, with participants preferring in-person counselling (Model 3 OR 0.72, *p* = 0.001; Model 4 OR 0.35 *p* = 0.001) and finger-prick testing (Model 3 OR 0.83 *p* = 0.054; Model 4 OR 0.17 *p* < 0.001). Participants remained indifferent regarding their preferences for self-administered versus provider-administered testing in both models.

## Discussion

Understanding the underlying preference structures that drive choices regarding HTC can help to explain why participants in this study made the choices they did in the context of the RCT and may help understand which service delivery model characteristics will facilitate uptake and which may act as barriers when HST is scaled up in a real-world setting. The main effects results of the DCE in these analyses among those in the intervention arm of the RCT show that, on average, preference structures of participants do not favour the attributes of oral HST over those of provider-administered finger prick testing already offered at North Star Alliance roadside wellness centres. This is a similar finding to our previous analysis which included the entire sample, both those in the intervention and those in the control arms [38]. Most notably, participants preferred in-person counselling to telephonic counselling, testing at a roadside clinic to testing at their office, and unsurprisingly, free testing and shorter testing times. Surprisingly, participants were indifferent between a free test and a test they were paid a small amount of money to use. Thus, the preference is for testing not to be linked to money in either direction. Also somewhat surprising, on average, participants were indifferent between oral and finger-prick testing, and between self- and provider-administered testing. These results suggest that, on average, HST may be an attractive option for truck drivers if testing time is perceived to be shorter [13], there is still a general preference for the HIV testing method already used in these clinics, which includes free, in-clinic testing that takes about 40 min with in-person counselling and support.

However, looking at the preferences of the sample as a whole, masked important differences in preferences between individuals or groups within the sample that might explain the diversity in HIV testing decisions participants made when offered choices.

### Stated preferences and actual choices in a clinical setting

When offered an actual choice between HIV tests at T1 (baseline), more than half the participants in the intervention arm opted for an oral self-test over a provider-administered finger prick test, which appears to contradict the DCE main effect results, although it is consistent with previous studies showing that the acceptability of HST is relatively high [13, 31]. To understand how the preference structures from the DCE analysis can help us understand the revealed preferences from the choices made in the RCT, we stratified the DCE analysis by test selected at T1 and found differences in preference structures between those who chose an oral self-test and those who chose a provider-administered finger-prick test at T1. The primary characteristic from the DCE that seems to be driving choice of test in the RP is the type of test (oral versus finger-prick). Participants who chose the self-test at T1 had a strong preference for oral-testing as opposed to finger prick testing in the DCE, while those who chose a provider-administered test at T1 preferred a finger prick test in the DCE. Fear of needles has been identified as a barrier to HIV testing for some people [13, 42], which may be a reason why some participants showed a preference for oral-testing. For those who showed a preference for a finger-prick test over an oral test, this may be an indication that they are not afraid of needles, but perhaps also that there may be a lack of knowledge or trust in new diagnostic technologies that are emerging – in this case, oral-testing [13, 35]. In the choices on offer at T1, all participants received in person pre-test counselling and all participants except for the few that chose to take a test home received in-person post-test counselling and support. Those who took a self-test kit for home use received post-test counselling over the phone. The DCE revealed that those who chose a provider-administered test had a strong preference for in-person counselling, while those who chose to self-test (some of whom took a test kit for home use with telephonic post-test counselling) had no preference regarding the type of counselling. This may be an indication of differences in the perceived utility gained from the personal interaction and support available from counsellors at healthcare facilities, affirming similar assertions made in previous studies [13, 43]. Other studies have also found that in different settings, HST under the supervision of a healthcare provider was preferred over HST without supervision, a further indication of the importance of in person support [44, 45].

Importantly, there were no differences in preferences regarding who administers the actual test (provider or self). Thus, the self-administration aspect of the self-test may not be so important, and it could be that offering provider-administered oral HIV testing would have a similar or perhaps greater impact on HIV test uptake than oral self-administered HIV testing. However, it could also be that for certain truck drivers, the self-administered aspect is important, but we did not identify the subgroup for which this was the case (i.e. perhaps we need to further stratify among those who chose the self-test on efficacy or some other variables that might distinguish for whom the administrator of the test (self or provider) is important.

In all analyses, cost was found to be a strong driver of choice, with participants preferring a free test to one they would have to pay for. Ensuring that HIV testing continues to be offered free of charge to truck drivers in Kenya, regardless of the type of test on offer, is likely to be important for facilitating uptake. Making test kits available for purchase is unlikely to be successful in increasing uptake of HIV testing among truck drivers based on current preference structures[13, 43]. The DCE results show that offering small financial incentives to test is also unlikely to significantly alter the decision to test, suggesting that participants prefer HIV testing to be unlinked to money in either direction.

Finally, the location of testing at T1 was the same for all participants, except for the 11 participants who elected to take a self-test for use outside the clinic, a number too small for examination as a separate subgroup and therefore included with those who self-tested in the clinic. Preference structures regarding location in the DCE were not found to be highly significant, with the exception of a preference to test in a roadside clinic over the office. This was somewhat unsurprising, as most participants had tested at roadside wellness centres previously and participants were recruited from roadside wellness centres. Previous research has shown that in some contexts, door-to-door home testing for HIV has been associated with increased uptake of testing amongst “hard-to-reach” men [45]. In this study, we could not look separately at the preferences of those who took a self-test for home use at baseline or follow-up due to small numbers, but these participants might have different preferences to those who self-tested in the clinic. Exploring the underlying preference structures and particularly focusing on location of testing in such subgroups as well as at truck drivers that do not use roadside wellness centres and those who are uncomfortable with clinical environments to see how they differ could be an important avenue for future research.

### Stated preferences and choices made in a real-world setting

In interpreting the results of this analysis, it is important to be cognisant of the context in which the data were collected, with choices made within the context of a research study by truck drivers who use North Star facilities to access primary healthcare. Therefore, we cannot assume the same choices would have been made in a real-world setting by all truck drivers. However, the testing amongst participants in our sample during follow-up was considerably closer to a real-world setting than the testing at baseline because the participant had to seek out testing, be it provider-testing in a clinic or pick-up of a self-test kit for home use. A comparison of testing at T1 and T2 could provide a more accurate indicator of the true effect when taken to scale.

Ten participants (7%) returned to self-test at T2, while a further eight (5%) returned for a self-test and also received the standard provider-administered testing compared to the 62 participants (44%) who returned to a clinic – which may or may not have been a North Star clinic – to receive provider-administered testing. This was substantially lower than the 95 participants (60%) who took a self-test at T1. Although the sample size of participants opting for a self-test at T2 was too small to determine drivers of choice in the DCE, there are several possible reasons for this result in relation to the preference structure patterns identified. Participants at T1 were already in the clinic with a healthcare worker, and spent time answering the initial questionnaire and engaging with the idea of oral HST through a demonstration by a healthcare worker before making a choice about HIV testing. Thus, rather than reflecting their true preference structures, choices at T1 may have in part been influenced by the setting in which the choice was offered, making them more likely to accept oral HST than at T2 – a finding akin to phenomena such as the Hawthorne Effect or social desirability bias often observed in health and behavioural research [20, 21]. Another possible explanation is that participants had little or no experience with oral HST at T1 and used the study to test a new diagnostic technology, rather than because is best aligned with preferences – almost 90% of those choosing to self-test at T1 cited one of their reasons as “being curious” about a new testing diagnostic [31] It is also possible that because HST was not widely available in Kenya at the time of this research, participants were not able to access self-testing kits from the facilities where they tested during the follow-up period. In any of these cases, more research will be required to understand how best to roll out HST in a way that will have a positive impact on demand and uptake of testing services.

When scaled up in a real-world setting, participants may be faced with an increased number of trade-offs when choosing between HST and traditional in-clinic provider-administered models of testing, depending on the models used for scale-up. For example, participants may have to trade off the in-person counselling and support they receive from healthcare workers when going to test at a clinic, and their preference for an oral test over a finger-prick test, available in self-testing kits (which might not be available for use inside a healthcare facility). The presence of these kinds of trade-offs in real-world scale-up settings may make it more difficult for some people to choose one service delivery model over another [17]. Offering an increased range of options may be a strategy to ensure increased uptake of HTC, for example, offering provider-administered oral-testing in a clinical setting, or offering both oral and finger-prick self-test kits that participants may take home. In this study, those who returned for a provider-administered test at T2 and those choosing a provider-administered test at both T1 and T2 had a clear preference for both in-person counselling and a finger-prick test. In this case, there is no trade-off in the provider-administered testing offered at roadside wellness centres and these clients are likely to continue to use existing services, rather than switch to HST.

Finally, some participants in this study reported having returned to a clinic for both a self-test and a provider-administered test at T2. Although the number of participants returning was small – leading to underpowered analysis – if HST is to be rolled out, it will be important to investigate these choices more carefully, both quantitatively and qualitatively. Researchers and implementers should seek to understand whether participants were using provider-administered tests to confirm the self-test results; whether the self-test kits were used to test themselves or partners; or whether there were other important reasons for participants returning for different types of tests within the six-month follow-up period, such as the lack of availability of self-testing kits at “non-North Star” facilities in Kenya accessed by participants at the time this study was conducted [13, 42, 46].

### Limitations and future research

This study has several limitations. Although the sample size for this study was sufficient for the main effects analysis, we were underpowered for some of the analyses looking at subsets of the participants by HIV test selected, especially at T2 when few participants self-tested, precluding our ability to look at this subgroup. We also note that there may have been some error in self-reported measures, particularly around HIV testing during follow-up which may have been influenced by social desirability bias.

Participants were recruited from the waiting room of two clinics and, because of this and other eligibility criteria, they may differ in important ways from other truck drivers such as those who do not access roadside wellness centres. This is an important limitation because, although it does not bias the results of our analysis, it limits the generalisability of the study findings to the population of truck drivers more broadly. Here, we acknowledge that there are likely some key areas where we might expect to see differences in preference structures between those truck drivers who attend clinics and who do not, but we believe that the results of this analysis remain important, given the large numbers of truck drivers who are already attending North Star Alliance clinics and the increasing visibility and acceptability of these facilities within the transport sector in Africa. Failure to find significant differences in the preferences regarding testing location and who administers the test might be because all participants had come to a roadside wellness centre, many for HIV testing. These individuals did not find clinic location or provider-administered testing (the only testing option offered at these clinics outside of this study) as barriers to seeking testing since many of them were seeking testing or other services at a clinic. If we included truck drivers who do not access clinic services, we might have found a stronger preference for testing outside of the clinic or self-administered testing.

It should be noted that self-testing kits were only available at the eight North Star Alliance clinics in Kenya. Thus, to self-test during follow-up, participants had to access one of these clinics, while they could access provider-administered testing at any North Star Alliance clinic in any country, as well as numerous other clinics that offer HIV testing. It could also be that, despite being told in-person once and sent an SMS reminder, participants did not fully understand that they could access self-testing kits at North Star Alliance clinics during follow-up or they thought that they could only obtain self-test kits at the same clinic where they were recruited into the study, which might not have been convenient.

For future research on this topic, a larger sample size would increase the power to detect significant differences in sub-group analyses. Additional subgroups might also be explored as HST preferences may align more closely by comfort in clinic settings, preferences for couples testing, or self-efficacy. Purposively sampling truck drivers who have never tested, a sub-group that was particularly small in this analysis, might also reveal a different HIV testing preference structure.

## Conclusion

This study used stated preference data to enrich the revealed preference data within a clinical trial setting, helping to explain some of the underlying reasons for the choices participants made. The analysis showed that the option of oral HST is well aligned with the preferences of some truck drivers – particularly those whose revealed preference was for HST – but that this was driven by a preference for an oral test over a finger-prick test, rather than a self-administered test over a provider-administered test. Those truck drivers with a revealed preference for provider-administered testing had stated preferences that were more closely aligned to the characteristics of the existing testing services already available at roadside wellness centres. The stated preference data suggest that this was driven primarily by a preference for in-person counselling and a finger-prick test, an indication that many truck drivers value the in-person support offered by health care workers and trust blood-based tests more than oral tests, a finding consistent with previous work [47, 48].

From a programmatic perspective, this study shows that the services provided by the North Star Alliance at their roadside wellness centres are already well-aligned with the average preferences of the truck drivers who come through their doors. Those who did prefer HST were likely to do so because of the HST kits offered an oral test (as opposed to a finger prick test), rather than that they preferred to administer the test themselves. In considering the possibility of expanding service delivery models, North Star Alliance should not consider abandoning the current models in favour of HST, but rather offer HST kits as an additional service, with the option for truck drivers to self-test with a healthcare provider supervising as well as take a test kit for home use. Offering an oral provider-administered HIV test as opposed to the finger prick test for their rapid assessment might also increase testing rates as it would reach those who fear blood or needles. Providing a wider range of options for HIV testing is likely to increase the reach and impact of this important network of roadside wellness centres. Further, offering HST kits through non-clinic venues, such as outreach at truck stops, something the North Star Alliance is already doing to make provider-administered HIV testing more accessible, may appeal to truck drivers who do not access clinics, and could potentially encourage partner or couples testing. A comprehensive understanding of preferences and other demand-side factors will be vital in ensuring that the way in which oral and self-testing are introduced and scaled up actually translates into increased uptake, not only among truck drivers who already access healthcare facilities, but importantly among those who are not reached through existing HIV testing services.

## Abbreviations

DCE: Discrete choice experiment
HST: HIV self-testing
HTC: HIV testing and counselling
OR: Odds ratio
RCT: Randomised controlled trial
RP: Revealed preference
SD: Standard deviation
SP: Stated preference
